# Successful embolisation of intrahepatic portosystemic venous shunt using AMPLATZER Vascular Plug II

**DOI:** 10.1259/bjrcr.20160061

**Published:** 2016-12-19

**Authors:** Akiko Tomiyama, Sota Oguro, Mayumi Kato, Hiromi Watanabe, Hiroya Yamazaki, Tatsuya Suzuki, Shinichi Tominaga

**Affiliations:** ^1^Department of Diagnostic Radiology, School of Medicine, Keio University, Tokyo, Japan; ^2^Department of Radiology, Saitama City Hospital, Saitama, Japan

## Abstract

An intrahepatic portosystemic venous shunt is a relatively rare abnormality that can cause encephalopathy owing to hyperammonaemia. Two patients with encephalopathy owing to intrahepatic portosystemic venous shunts were treated with transcatheter emboli sation using the AMPLATZER Vascular Plug II. Both patients achieved complete obliteration, which was confirmed on dynamic CT. Their symptoms that had been related to portalsystemic encephalopathy subsequently improved after the intervention. No short-term complications were observed in either patient. We recommend that the AMPLATZER Vascular Plug II be used for embolis ation owing to its superior safety and utility when compared with metallic coils or other liquid embolic materials.

A portal vein hepatic vein shunt (PV shunt) in the liver is a relatively rare abnormality^1,2^ that can cause hyperammonaemia and neurological symptoms.^2–4^ Previous studies reported little success when using conventional treatment with interventional radiological strategies.^2^ This is because the blood flow at the site of a PV shunt is rapid, and coils placed at the PV shunt sometimes migrate out of the target site.^5,6^

Recently, the AMPLATZER Vascular Plug (AVP) II (St. Jude Medical, Tokyo, Japan) has become available for clinical use. The AVP II is one of the biggest plugs and has been used for embolisation of relatively thick vessels. Use of vascular plugs is associated with significant cost savings if a single device can achieve complete occlusion.^7^ Paudel et al reported that transhepatic embolisation of congenital intrahepatic PV shunts using vascular plugs could be safely performed.^8^ However, percutaneous transcatheter embolisation of PV shunts in the liver using vascular plugs has not yet been reported. The present report describes two patients who underwent the percutaneous transcatheter embolisation of PV shunts in the liver using vascular plugs.

## Case report

### Case 1

An 83-year-old male with chronic hepatitis was noted to have a hepatic mass with early enhancement in the right lobe of the liver. A PV shunt was also noted close to the tumour on dynamic abdominal CT. The hepatic tumour was diagnosed as hepatocellular carcinoma by ultrasound-guided biopsy, and transcatheter arterial chemoembolisation was performed. This patient had mild encephalopathy, and the serum ammonia level was already increased to 104 μg dl^−1^ before the chemoembolisation. Hyperammonaemia worsened to 144 μg dl^−1^ and mild hepatic encephalopathy continued after treatment of the hepatocellular carcinoma. Amino acid solution (Aminoleban; Otsuka Pharmaceutical, Tokyo, Japan) and lactulose did not alleviate the hyperammonaemia. Therefore, transcatheter embolisation of the PV shunt was planned.

Procedure: The right femoral vein was punctured using ultrasound guidance, and a 6 Fr 11 cm sheath introducer was inserted. The right hepatic vein was catheterized using a 6 Fr 20 mm balloon catheter (SELECON, Terumo, Tokyo, Japan). Two pathways from the portal vein to the hepatic vein were seen on the preoperative dynamic CT. Since the right hepatic vein was dilated and because flow at the PV shunt was rapid, occlusion of the right hepatic vein was deemed impossible using the 20 mm balloon catheter. Therefore, only one pathway could be catheterized and balloon-occluded. After the balloon occlusion, the location of the PV shunt was confirmed using retrograde venography. One of the pathways of the PV shunt was embolized using 10 Interlock detachable coils (six 14 mm × 30 cm, two 14 mm × 20 cm and two 12 mm × 30 cm) (Striker, Tokyo, Japan) under flow control using the balloon catheter. Next, an attempt was made to catheterize the other pathway, but it was unsuccessful despite the use of several different preshaped catheters. Meanwhile, the patient was not able to remain still on the bed because of hepatic encephalopathy. Thus, embolisation of the right hepatic vein using an AVP II was planned.

**Figure 1.**
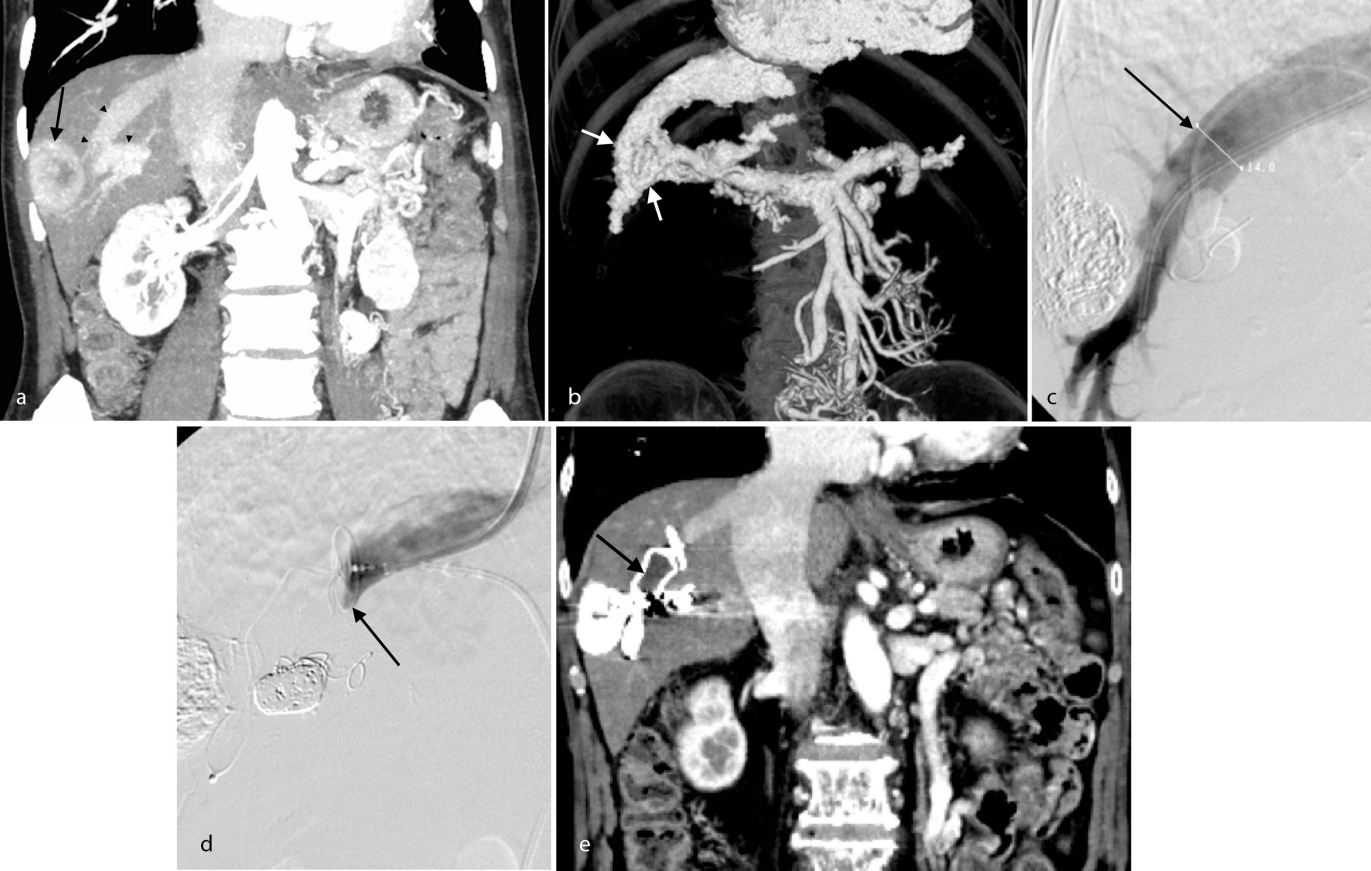
An 83 year-old male followed up for chronic hepatitis. (a) Coronal image of dynamic CT before chemoembolisation shows enhancing tumour (arrow) and a portosystemic shunt (arrowheads). (b) Volume rendering image based on abdominal dynamic CT showed a torturous shunt between the portal vein (P5) and the right hepatic vein (arrows). (c) Venography after balloon occlusion of one pathway of the portal vein hepatic vein shunt. The diameter of the right hepatic vein is 14  mm (arrow). The shunt was embolized using the Amplatzer Venous Plug II. (d) Venography after deploying the Amplatzer Venous Plug II; embolisation of the right hepatic vein is indicated (arrow). (e) Dynamic CT image 1 month after the procedure shows complete embolisation of the portal vein hepatic vein shunt (arrow).

After the right jugular vein was punctured using ultrasound guidance, a 9 Fr 11 cm sheath was inserted. A 9 Fr multipurpose-type catheter (Bright tip, Cordis Corporation, Miami, FL) was led into the right hepatic vein, and the AVP II (22 mm) was deployed at the right hepatic vein. The procedural time was 135 min. The next morning, the serum ammonia level decreased to 34 mg dl^–1^, and hepatic encephalopathy had improved. Successful embolisation of the PV shunt was confirmed on abdominal dynamic CT that was performed 2  months after the embolisation (Figure 1).

### Case 2

A 75-year-old female presented to the emergency room of our hospital after sudden onset of confusion and incontinence. She had no medical history of trauma, liver disease or loss of consciousness. Her serum ammonia level was elevated to 330 mg dl^–1^. Head CT showed no obvious cause for her confusion. Consequently, she was diagnosed with hepatic encephalopathy. Based on our experience with the case described above, an embolisation of the PV shunt using the AVP II was planned.

**Figure 2.**
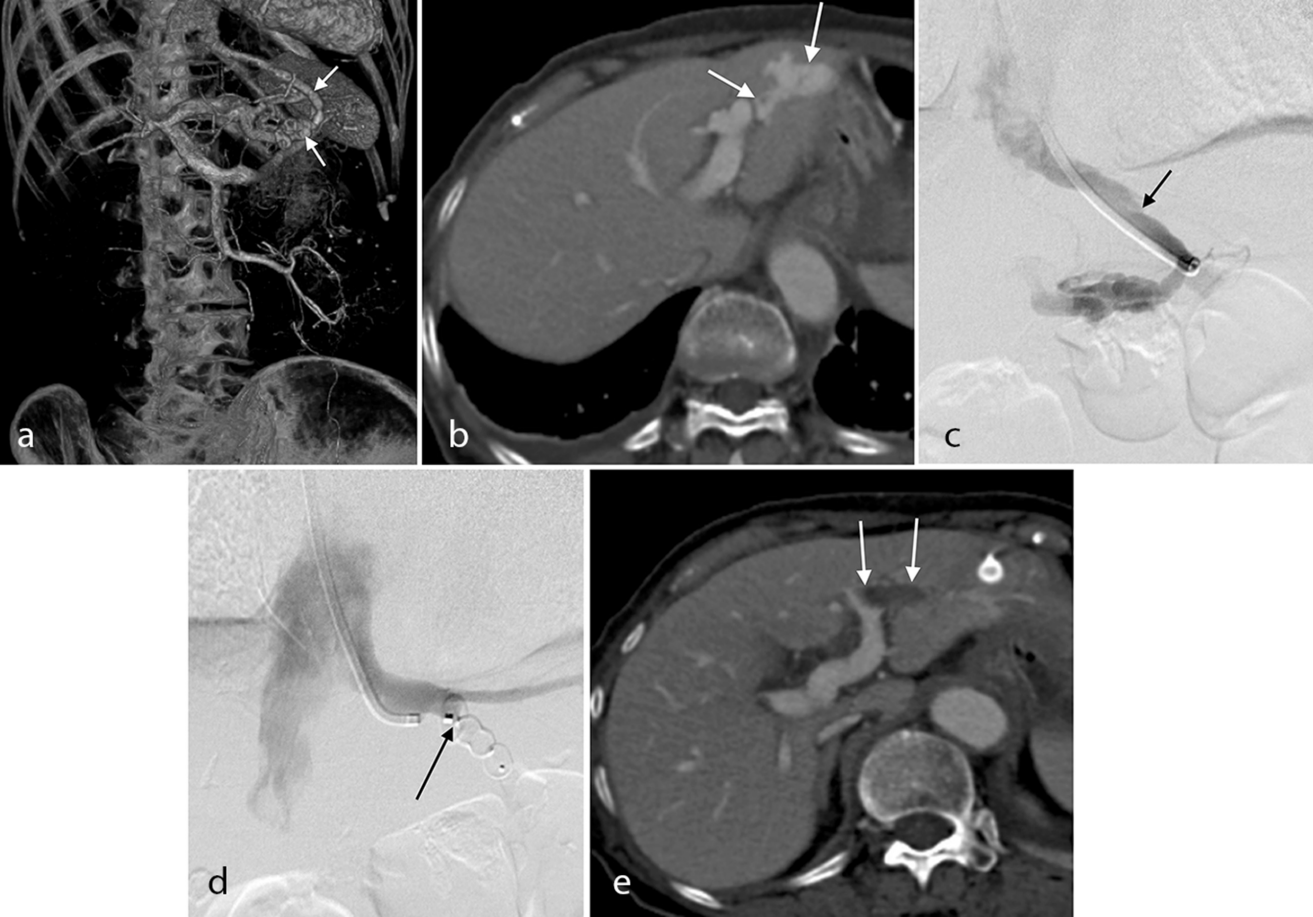
A 75-year-old female: (a, b) Pretreatment dynamic CT and volume rendering image based on dynamic CT shows a tortuous shunt between the portal vein (P3) and the left hepatic vein (arrows). (c) Venography after catheterisation of the left hepatic vein. The diameter of the left hepatic vein is 7 mm (arrow). (d) Venography after deploying the AMPLATZER Vascular Plug II: embolisation of the left hepatic vein is shown, and embolisation of the right hepatic vein is indicated (arrow). (e) Dynamic CT 1 month after the procedure shows complete embolisation of the portal vein hepatic vein shunt (arrows).

Procedure: The right jugular vein was punctured under ultrasound guidance, and a 9 Fr 11 cm sheath was inserted. Then, a 9 Fr multipurpose type catheter (Bright tip, Cordis Corporation) was advanced to the left hepatic vein. Digital subtraction venography showed the location of the PV shunt. Then, a 14 mm AVP II was deployed at the left hepatic vein through the catheter. Retrograde venography of the left hepatic vein indicated obstruction of the PV shunt. The procedural time was 45 min. The next morning, the serum ammonia level had decreased to 30 mg dl^–1^, and the hepatic encephalopathy had improved markedly. The success of embolisation of the PV shunt was confirmed on abdominal dynamic CT performed 1 month after the embolisation (Figure 2). The hepatic encephalopathy did not reappear, and the ammonia level did not increase to more than 80 μg dl^–1^ up to 5 months later in both cases.

## Discussion

Intrahepatic PV shunts can be treated using surgical ligation or with embolisation via the percutaneous transhepatic portal vein approach or transileocolic vein approach under small laparotomy.^9,10^ PV shunt embolisation using an AVP II from a percutaneous transjugular or transfemoral venous approach has not been reported, but Lee et al^10^ previously reported embolisation of a PV shunt using an AVP II via a transhepatic approach. First, they tried to approach the portal side of the PV shunt from the percutaneous transjugular approach, but it was impossible. Therefore, they changed to a percutaneous transhepatic approach. They then deployed the AVP II at the portal vein side of the PV shunt. In our strategy, the occlusion target was not the sac of the PV shunt but the venous side of the PV shunt. After deploying the AVP II at the venous side of the PV shunt, occlusion of the PV shunt itself could be expected, and if the PV shunt did not occlude, the sac of the portosystemic shunt could be approached and occluded via a percutaneous transhepatic approach.

Transcatheter embolisation of the PV shunt is an excellent method because it is relatively less invasive.^11^ However, coil migration can occur in the context of a portal venous shunt^6^ with deviation of the embolic materials to the systemic circulation. When embolizing the PV shunt, the flow at the shunt point is frequently rapid, and embolisation of the PV shunt under balloon occlusion is therefore sometimes used.^12^ In the first case described in this report, one of the pathways of the PV shunt was successfully embolized using metallic coils under balloon occlusion, but the other pathway could not be catheterized, subsequently necessitating the use of an AVP II for embolisation. When deploying the AVP II at the venous side of the PV shunt, the shunt vessels were occluded owing to the blood remaining, and this produced thrombus within the AVP II. However, when the blood flow of shunts is too fast or when the patient has disseminated intravascular coagulation, thrombus cannot be produced in the AVP II.^7^ In the first case, there was no absolute proof that the PV shunt would be embolized by the AVP II. However, if the embolisation of the PV shunt was not achieved, a second procedure could have been performed using a transdermal transhepatic portal vein approach.^8^ Even if the AVP II deployed from the venous side could not embolize the PV shunt, the AVP II can act as an anchor when a transdermal transhepatic portal vein approach is used. Therefore, the risk of deviation of the embolic materials to the systemic circulation is extremely low.

Chevallier et al^13^ categorized intrahepatic portosystemic venous shunts (IPSVS) into four types: (1) patent paraumbilical veins in the liver, commonly encountered in patients with portal hypertension; (2) single or multiple shunts between an intrahepatic portal branch and a hepatic vein located in one of two adjacent liver segments; (3) single or multiple shunts between an intrahepatic portal branch and a hepatic vein located in non-adjacent liver segments; and (4) any tubular communication between the right portal branch and the inferior vena cava. The cause of IPSVS is controversial.^6,13–16^ The method used in this report could conceivably be applied for the management of Type 2 or Type 3 IPSVS.

The limitations of this study include its small sample size and the short follow-up period that did not allow adequate assessment of the effect of embolisation. Embolisation by an AVP II is possible only when the catheter can access the PV shunt.

In conclusion, the PV shunts in the two cases described in this report were successfully embolized using an AVP II without any short-term adverse events. Embolisation of the PV shunt using an AVP II was easy, and the procedural time was short. We conclude that percutaneous transcatheter embolisation using vascular plugs is strongly recommended for embolisation of intrahepatic PV shunts.

## Learning points

1. Embolization of the PV shunt using an AVP II can be easily performed in short procedure time.
2. Percutaneous transcatheter embolisation using vascular plugs is strongly recommended for embolisation of intrahepatic PV shunts.

## Consent

Written informed consents were obtained from all subjects for publication of this case report and accompanying images. A copy of the written consent is available for review upon request.
